# Deep-Sea Benthic Footprint of the Deepwater Horizon Blowout

**DOI:** 10.1371/journal.pone.0070540

**Published:** 2013-08-07

**Authors:** Paul A. Montagna, Jeffrey G. Baguley, Cynthia Cooksey, Ian Hartwell, Larry J. Hyde, Jeffrey L. Hyland, Richard D. Kalke, Laura M. Kracker, Michael Reuscher, Adelaide C. E. Rhodes

**Affiliations:** 1 Harte Research Institute for Gulf of Mexico Studies, Texas A&M University-Corpus Christi, Corpus Christi, Texas, United States of America; 2 Department of Biology, University of Nevada-Reno, Reno, Nevada, United States of America; 3 National Centers for Coastal Ocean Science, National Oceanic and Atmospheric Administration, Charleston, South Carolina, United States of America; 4 National Centers for Coastal Ocean Science, National Oceanic and Atmospheric Administration, Silver Spring, Maryland, United States of America; Universidade Federal do Rio de Janeiro, Brazil

## Abstract

The Deepwater Horizon (DWH) accident in the northern Gulf of Mexico occurred on April 20, 2010 at a water depth of 1525 meters, and a deep-sea plume was detected within one month. Oil contacted and persisted in parts of the bottom of the deep-sea in the Gulf of Mexico. As part of the response to the accident, monitoring cruises were deployed in fall 2010 to measure potential impacts on the two main soft-bottom benthic invertebrate groups: macrofauna and meiofauna. Sediment was collected using a multicorer so that samples for chemical, physical and biological analyses could be taken simultaneously and analyzed using multivariate methods. The footprint of the oil spill was identified by creating a new variable with principal components analysis where the first factor was indicative of the oil spill impacts and this new variable mapped in a geographic information system to identify the area of the oil spill footprint. The most severe relative reduction of faunal abundance and diversity extended to 3 km from the wellhead in all directions covering an area about 24 km^2^. Moderate impacts were observed up to 17 km towards the southwest and 8.5 km towards the northeast of the wellhead, covering an area 148 km^2^. Benthic effects were correlated to total petroleum hydrocarbon, polycyclic aromatic hydrocarbons and barium concentrations, and distance to the wellhead; but not distance to hydrocarbon seeps. Thus, benthic effects are more likely due to the oil spill, and not natural hydrocarbon seepage. Recovery rates in the deep sea are likely to be slow, on the order of decades or longer.

## Introduction

The Deepwater Horizon (DWH) accident in the northern Gulf of Mexico occurred on April 20, 2010 at a water depth of 1525 meters, in Mississippi Canyon Block 252, releasing an estimated 4.6 million barrels ( = 193 million U.S. gallons, or 731 million liters) of oil to the Gulf of Mexico through July 15, 2010 [1]. While oil-budget estimates indicate a majority of the oil had been removed by cleanup operations and other natural mechanisms [2], up to 35% of the hydrocarbons were trapped and transported in persistent deep-sea plumes [3]. Thus, the DWH blowout actually presents two incidents: the familiar buoyant oil spill with surface effects of short residence times, and the novel deepwater plume with chronic subsurface effects that suppress population recovery of exposed animals [4]. In addition, there were likely mid-water impacts to plankton and a variety of mid-water species. Oil in the deepwater plume was transported to deepwater sediments via multiple pathways, e.g., direct sinking of oil, adsorption of small oil droplets (alone or mixed with dispersant) onto suspended organic and inorganic particles in marine snow, incorporation into sinking copepod fecal pellets in either surface or sub-surface layers, onshore-offshore transport of oil-laden particles, sinking of heavier oil by-products resulting from the burning of oil, or settling of oil-mud complexes resulting from the injection of drilling muds during top-kill operations [5]. Heavy metals such as barium are components of drill cuttings, drill fluids, and other containment fluids commonly used during offshore oil-drilling operations [6], [7] and were likely released and deposited to the bottom during the blowout event.

Contaminants transported to the seafloor may pose risks to benthic fauna, particularly those living within or in close association with bottom substrates and unable to avoid exposure due to their relatively sedentary existence. Potential ecosystem service losses are of concern because these fauna serve vital functional roles in the deep-sea ecosystem including biomass production, sediment bioturbation and stabilization, organic matter decomposition and nutrient regeneration, and secondary production and energy flow to higher trophic levels [8], [9]. In many places, the deep-sea benthos represent important reservoirs of marine biodiversity [10], [11], [12], [13]. High benthic species diversity has been reported for the Gulf of Mexico with a maximum on the mid to upper continental slope at depths between 1200 to 1600 meters [14], [15], which coincides with depths of the DWH well site and potential zone of exposure. The loss of benthic biodiversity is correlated to an exponential decline in deep-sea ecosystem functioning [8]. Because deep-sea benthic communities are diverse, are a critical part of the foodweb base, play a key role in carbon cycling, affect productivity, are sensitive to perturbations, and are at risk to contaminant exposure, it is important to determine the effects that the DWH blowout might have had on these natural resources.

## Methods

After the MC252 well was capped on 15 July 2010, an Implementation Plan for subsurface monitoring was developed by the Unified Area Command to assess the presence of oil posing a threat to public health or the environment [5]. The Plan focused on sampling deep-sea sediments where oil may have migrated and where gaps in previous sampling efforts existed. Two field missions were conducted on the *R/V Gyre* (September 16 through October 19, 2010) and *R/V Ocean Veritas* (September 24 through October 30, 2010). While 170 stations were sampled, 68 stations located from 0.5 km to 125 km from the wellhead and at water depths ranging from 76 m to 2767 m were designated as priority stations and analyzed for the current study. Stations were located along a suspected gradient of contaminant effects where 16 of the stations were arranged in a “bulls-eye” design. This survey design was used because transects extending in radial patterns from the source of contamination and the statistical analysis of such designs is well known [16].

Sediment samples were collected using an OSIL multicorer, which takes 12 simultaneous cores from a single deployment at each station. The cores are 10 cm inner diameter and 60 cm in length. Samples were collected in a multivariate design for each station. Three cores were set aside for benthic macrofauna, one core was used for benthic meiofauna, one core was used for measuring oil and other drilling related contaminants, and one core was used for basic habitat characteristics (sediment grain size and sediment water content).

This study was performed in the US Exclusive Economic Zone (EEZ), and not on any private land. These are not protected lands or waters. No permission for taking samples was required, nor was it sought. These studies did not involve any endangered or protected species.

Macrofaunal samples were processed by extruding cores into two vertical sections (0–5 cm and 5–10 cm) immediately after collection. Each section was preserved in the field in 4% buffered formalin with Rose Bengal, sieved in the laboratory on a 0.3-mm mesh screen, and transferred to 70% ethanol; and animals from each of the above samples were counted and identified typically to the family level or higher.

Meiofaunal samples were collected by immediately subsampling with a smaller core (5.5 cm inner diameter). The subcores were extruded into two vertical sections (0–1 cm and 1–3 cm); relaxed in the field in 7% MgCl_2_ and preserved in 4% buffered formalin with Rose Bengal, sieved in the laboratory on a 0.042-mm mesh screen, and transferred to 70% ethanol; and sorted animals from each of the above samples were counted and identified to the lowest possible taxonomic level, which generally was order level or higher.

Diversity was calculated for macrofauna identified to the family level and meiofauna identified to higher taxonomic levels ranging from phylum to order. Using higher taxonomic levels in diversity studies is twice as rapid and has been shown to yield results similar to those using species level diversity indices to assess pollution status around oil and gas platforms in the Gulf of Mexico [17]. Species diversity was calculated by replicate using Hill's diversity number one (N1) [18]. It is a measure of the effective number of species in a sample, and indicates the number of abundant species [19]. It is calculated as the exponentiated form of the Shannon-Weiner H′ diversity index, N1 = е^H′^. As diversity decreases, N1 tends toward 1. Hills’ N1 was used because it is easier to interpret than most diversity indices.

Chemical contaminant and sediment grain size data collected in the same multicorer drops as the infauna. Contaminant measurements were made on the top 3 cm of sediment. Data were downloaded from http://files.noaanrda.org/on 2 April 2012. This is the same data set reported on in the UAC (2010) report [5]. The data is also available at http://www.ncddc.noaa.gov/activities/healthy-oceans/jag/data/and http://www.restorethegulf.gov/release/2010/12/16/data-analysis-and-findings. Methods for the chemical analyses are also described in the report and at http://www.nodc.noaa.gov/deepwaterhorizon/ship.html.

GIS shape files were obtained externally. The bathymetry map is courtesy of Bill Bryant (TAMU, retired). The seep map portrays all known acoustic 3D seabed anomalies for the deep Gulf of Mexico compiled by the Bureau of Ocean Energy Management, Regulation, and Enforcement (BOEMRE). The seep map was completed by Bill Shedd and Jesse Hunt (prior to his retirement) in the Gulf Of Mexico Resource Evaluation section. Over 21,000 geological features are described in the seep map, but many of them maybe relict, inactive seeps. The seep map was downloaded on 8 November 2011 from http://www.boemre.gov/offshore/mapping/SeismicWaterBottomAnomalies.htm downloaded 8 November 2011, but the linked moved to http://www.boem.gov/Oil-and-Gas-Energy-Program/Mapping-and-Data/Map-Gallery/Seismic-Water-Bottom-Anomalies-Map-Gallery.aspx and was downloaded 14 May 2013.

All biotic and chemical variables (X) were log transformed using ln (X+1), except the N1 diversity index, which is already a log transformation. After transformation, all variables were standardized to a normal distribution with a mean of 0 and variance of 1 using the PROC STANDARD module contained in the SAS software suite. Raw and transformed data is provided in supplementary materials (Table S1).

Principal components analyses (PCA) was used to classify the biological and environmental variables. The PCA is a variable reduction technique that can be used to reduce a large number of variables to a reduced set of new variables, which are uncorrelated and contain most of the variance in the original data set. PCA was performed using the PROC FACTOR module contained in the SAS software suite. The FACTOR analysis was run using the PCA method on the correlation matrix. A multiple linear regression analysis was also performed using PROC REG using abiotic variables to explain patterns in biotic variables and to evaluate the significance and direction of their associations.

The PC1 station scores were plotted in ArcMap 9.3.1 to illustrate the spatial extent of DWH impacts. Jenks natural breaks optimization (Goodness of Variance Fit) was chosen to separate PC1 into five classes, because this model forms classes based on minimum within-class variance and maximum between class variance [20]. As such, the model successfully separated PC1 into five natural classes over the range of PC1 scores where the largest positive values of PC1 (red and orange circles) represented stations with highest chemical loads and nematode to copepod (N:C) ratios, and lowest diversity indices (N1), all of which indicate DWH impacts. Conversely, the large negative PC1 scores represented high diversity and low chemical loads representative of natural background conditions (yellow/green, and green circles). Intermediate PC1 scores (yellow circles) were less than the median and are therefore not considered to be impacted by the DWH oil spill.

Interpolated maps were constructed in ArcMap Geostatistical Analyst based on PCA results (Factor 1 scores – unrotated). Kriging geostatistical techniques were used to interpolate the value of the random filed to predict the footprint on the map because the data are spatially autocorrelated. An advantage of the kriging method is that it incorporates local variation to model the spatial behavior of an event such as the impacts around the wellhead. Ordinary kriging incorporates semivariogram analyses that model the underlying spatial pattern to predict values at unsampled locations. The Geostatistical Analyst settings used in our analysis were: Final model = Gaussian function, number of lags = 12, lag size = 1200, nugget = 0.651, neighbors = 12 with a minimum of 2, RMSE = 0.9. Interpolated surfaces were converted to vector polygons to calculate area.

## Results

**Figure 1:**
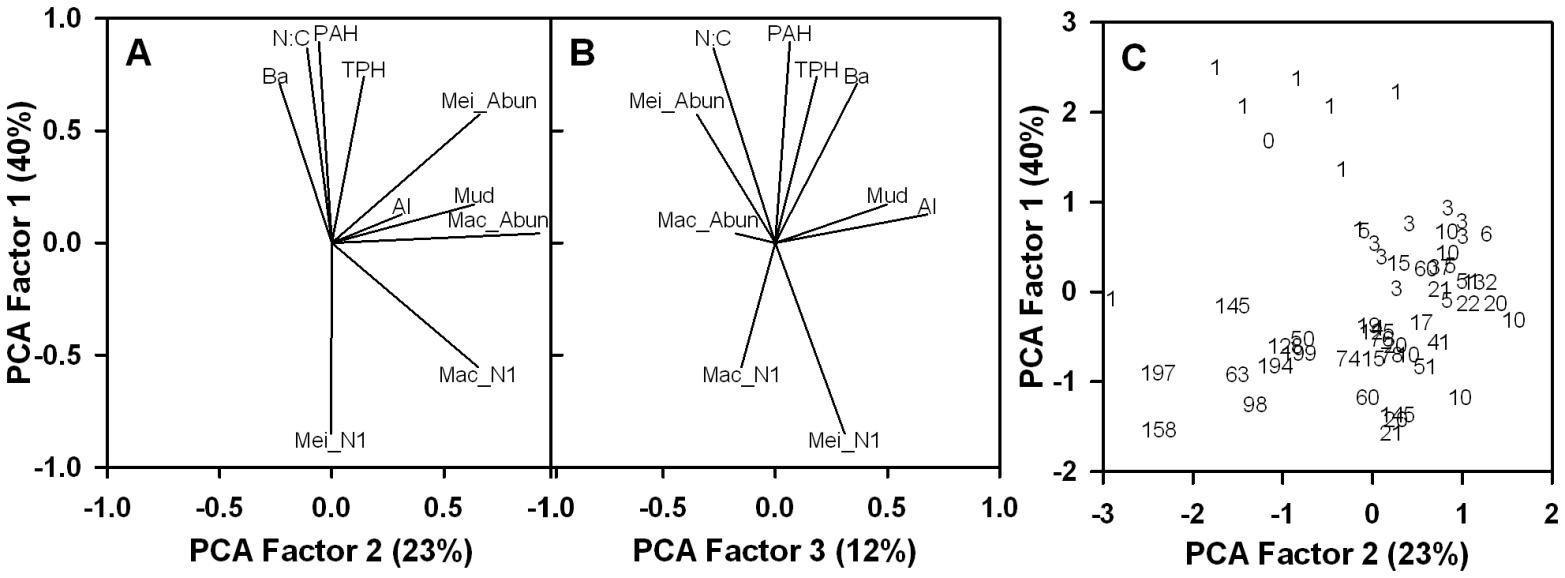
Principal components analysis of environmental and biological variables. (**A**) PC1 and PC2 vector loads for barium (Ba), polycyclic aromatic hydrocarbons (PAH), total petroleum hydrocarbons (TPH), percent mud content of sediment (Mud), aluminum (Al), nematode to copepod ratio (N:C), meiofauna abundance (Mei_Abun), macrofauana abundance (Mac_Abun), macrofauna Hill’s N1 diversity index (Mac_N1), and meiofauna Hill’s N1 diversity index (Mei_N1). (**B**) PC1 and PC3 vector loads. **(C)** PC1 station scores, where each station is labeled as distance in km from the wellhead.

Only the first three extracted orthogonal principal component (PC) factors had eigenvalues greater than 1 (Fig. 1A). DWH-derived contaminants were strongly associated with one another (Fig. 1A) and very highly loaded on PC1 (eigenvalue of 4.0), which explained 40% of the variability in the dataset. The second orthogonal variable, PC2 (eigenvalue of 2.3) explained 23% of the variability, and the third orthogonal variable (eigenvalue 1.2) explained 12% of the variability. Barium (Ba) is a common component of drilling muds and fluids and is typically associated with elevated levels of polycyclic aromatic hydrocarbons (PAH) and total petroleum hydrocarbons (TPH) around drill sites [7]. The sum PAH definition here is the PAH44 definition used by NOAA where the sum includes alkylated derivatives of the parent compounds (C1-, C2-, C3-, and C4-compounds) and some compounds with sulfur or oxygen substituted for carbons (thiophenes and furans). Consistent with ecological theory, when such contaminant concentrations were high, the nematode to copepod ratio (N:C) tended to be high and values of macrofauna Hill’s N1 diversity index (Mac_N1) and meiofauna Hill’s N1 diversity index (Mei_N1) tended to be low. PC1 represents the oil spill footprint.

The orthogonal axis, PC2 (eigenvalue 2.3), explained 23% of the variability in the data set and was related to positive associations between percent mud content (Mud) of sediment (grainsize <63 µm) and macrofauna abundance (Mac_Abun). PC2 represents additional benthic community characteristics that are related to water depth differences and the oil footprint (Table 1).

**Table 1:** Pearson correlation coefficients (and probability levels) for the principal component station scores and station locations with respect to depth (m), distance from the wellhead (km), and distance from seeps (km).

Station Location	Pearson Correlation (probability)		
	PC 1	PC 2	PC 3
**Wellhead**	−0.487 (0.0001)	−0.403 (0.0018)	−0.320 (0.0144)
**Seep**	−0.248 (0.0604)	−0.188 (0.1568)	−0.496 (<0.0001)
**Depth**	0.0456 (0.7339)	−0.435 (0.0006)	−0.217 (0.1022)

n = 58.

PC3 was significant (eigenvalue 1.2) and explained an additional 12% of the variance, it defines the natural background with mud and aluminum (Al) concentrations being correlated and the only two variables contributing positive loads. In contrast, macrofauna abundance contributed negative loads to PC3. PC3 represents the natural background of the deep-sea sediment grain size and Al content, both of which do not vary greatly in Gulf of Mexico sediments [7].

**Figure 2:**
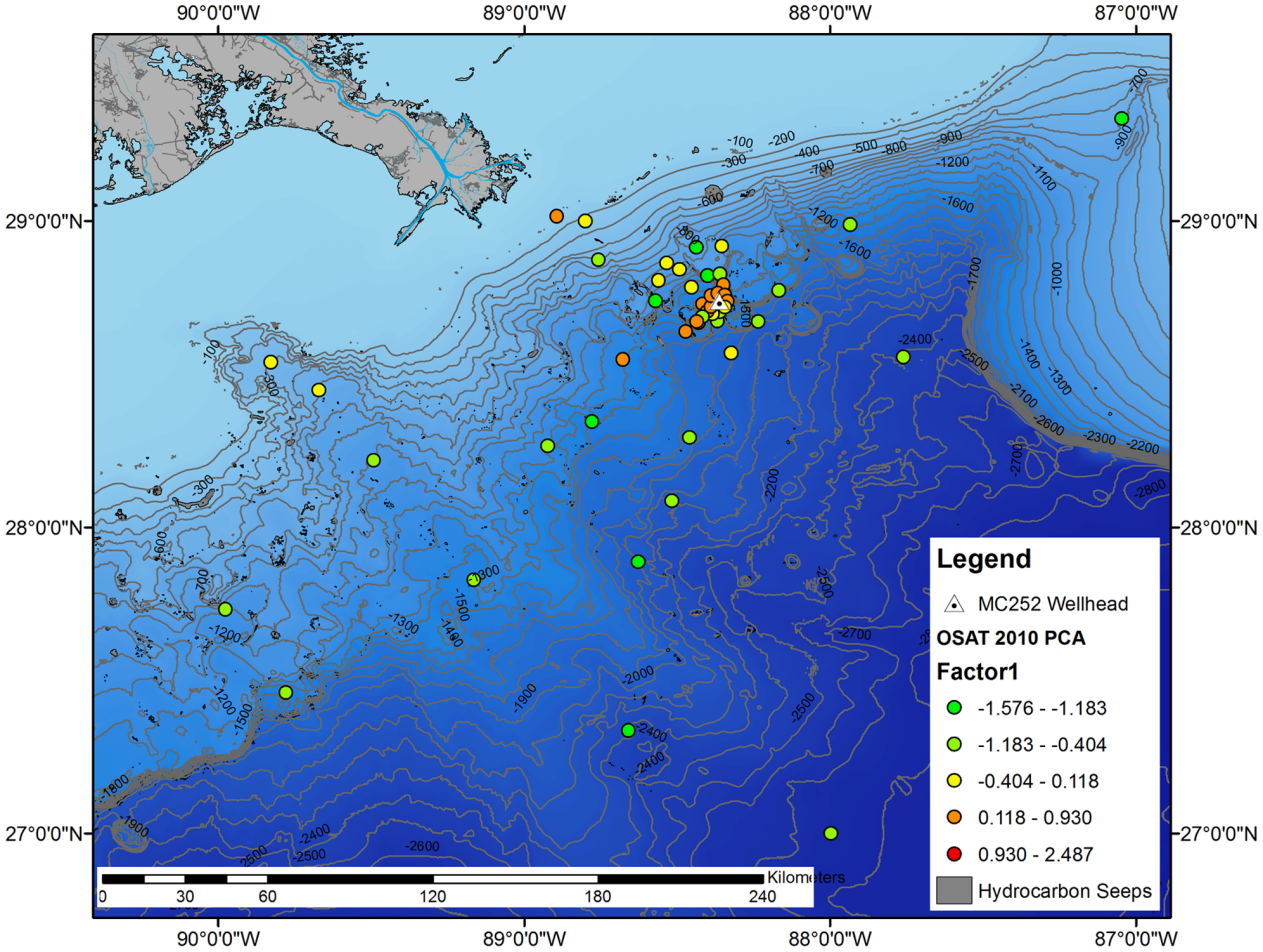
PC 1 station scores (Fig. 1) plotted as Jenks natural breaks. Map includes bathymetry in meters and locations of seeps.

Station scores for the new PC1 variable (the oil spill footprint) were classified into five natural breaks using the Jenks algorithm and mapped to determine the spatial distribution of the oil spill related impacts on deep-sea sediments (Fig. 2). These five groups were color-coded from the highest positive values (red dots) to the lowest negative values (green dots). The red and orange dots, which indicated strong and moderate impacts respectively, cluster mostly near the DWH wellhead. With one exception (a station 60 km to the northwest of the wellhead), the orange dots (moderate impacts) occur within 3 km of the wellhead in all directions and at several stations from 5–15 km away from the wellhead, especially to the southwest. An additional orange dot is found as far as 37 km to the southwest of the wellhead along the same isobath. Otherwise, only natural background values are found at the regional scale.

**Figure 3:**
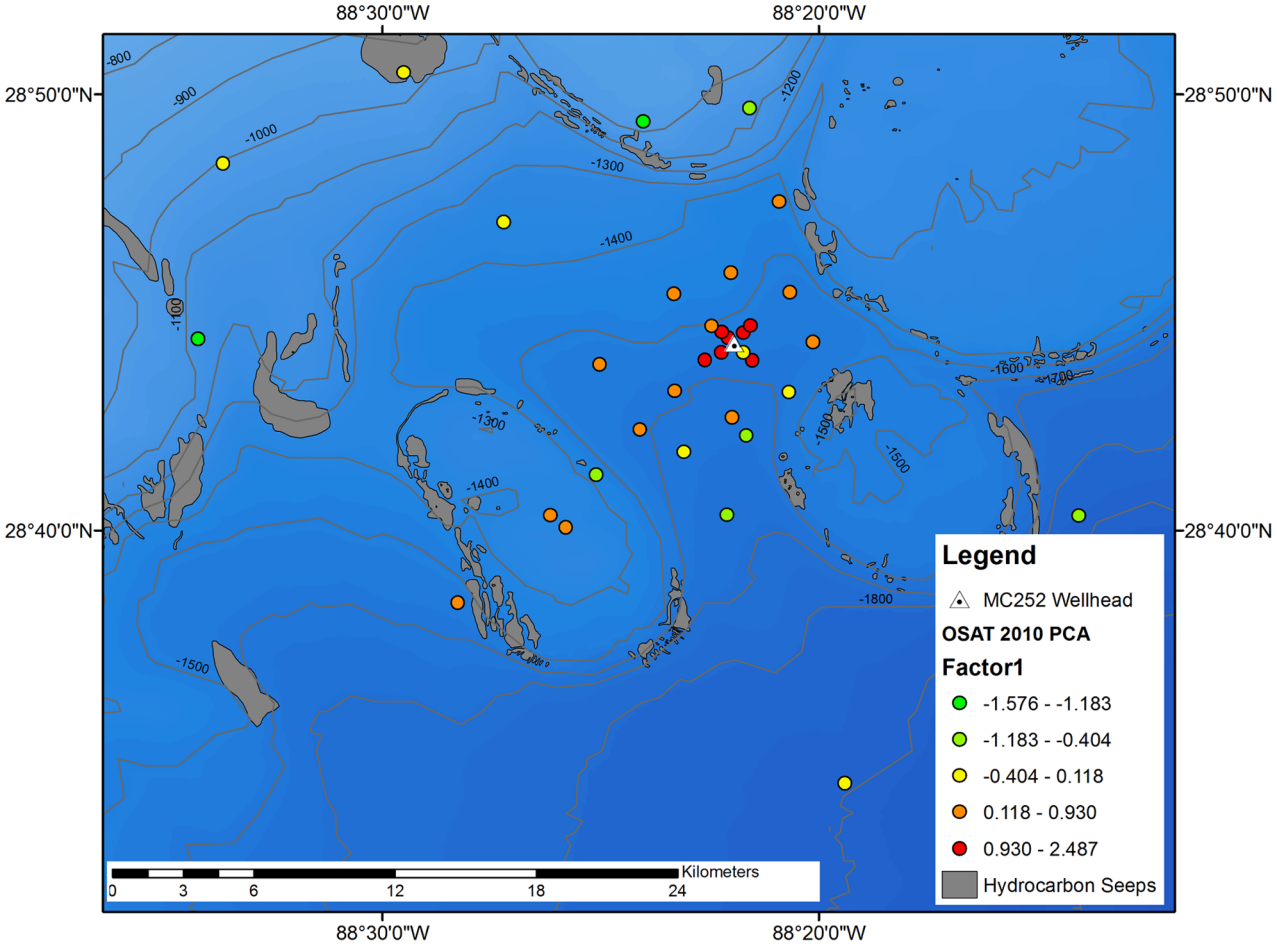
PC 1 station scores (Fig. 1) zoomed into the 40 km from the wellhead, and plotted as Jenks natural breaks. Map includes bathymetry in meters and locations of seeps.

Benthic community response in the five zones was strongest for the N:C ratio, which was 240.1% higher in the red zone than the overall sample mean, and decreased in each successive zone (Table 2, Figs 2–3). Because of the increase in nematodes, the total meiofauna abundance was highest in the red and orange zones. Meiofauna diversity and macrofauna diversity exhibited decreases in successive zones. Macrofauna abundance was lowest in the red zone, then increased to the yellow zone, but decrease in the green and light green zones.

**Table 2:** Percent change relative to overall mean for benthic community response in zones identified in Figs. 2–3.

Color	Zone	MacrofaunaAbundance	MeiofaunaAbundance	MacrofaunaDiversity	MeiofaunaDiversity	Nematode:Copepod Ratio
**Red**	1	−30.2%	43.2%	−53.7%	−38.3%	240.1%
**Orange**	2	17.6%	50.9%	−4.5%	−19.0%	20.0%
**Yellow**	3	25.4%	3.9%	14.5%	−2.4%	−31.3%
**Lt Green**	4	−13.3%	−43.7%	6.3%	16.4%	−57.5%
**Green**	5	−7.1%	−27.3%	11.9%	22.8%	−58.4%

Zooming in, all but two stations within 1 km of the wellhead have red dots indicating the highest degree of impacts in the immediate near-field zone (Fig. 3). Generally, there is a gradient in the groups with distance from the wellhead, indicating very subtle effects could be detected at very far distances, and the shape of the footprint is important. While moderate impacts (orange dots) extend out to about 6 km in various directions from the wellhead, they also extend along a narrower corridor approximately 37 km to the southwest. The southwest extension of the DWH footprint is consistent with the reported direction of the deep-sea plume of particulate, dissolved, and chemically dispersed oil along an isobath of about 1400 m. The shallowest station, located 60 km northwest of the wellhead at a water depth of 76 m, also showed evidence of a moderate impact. This isolated case is an exception to the above footprint of impacts within the near-field zone around the wellhead and farther-field sites to the southwest, but is consistent with the station’s location along the observed path of offshore-onshore movement of surface oil slicks that followed the blowout.

**Figure 4:**
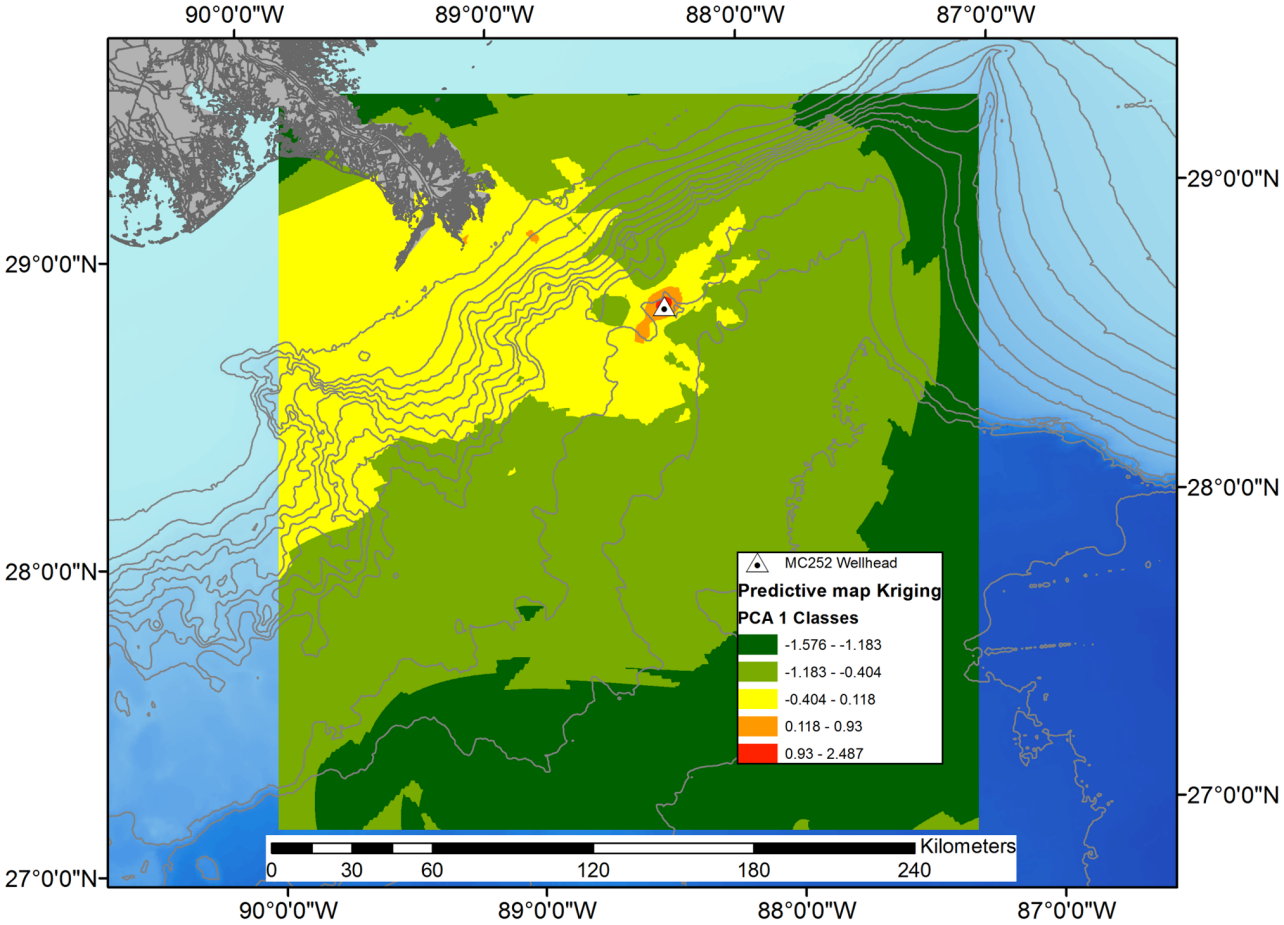
Interpolated area of deep sea impact based on PC1 station scores. The interpolated area shown covers 70,166 km^2^ of which 167 km^2^ (orange) are considered moderately impacted and 24 km^2^ (red) are considered severely impacted.

The footprint of the oil spill effects was modeled by Kriging the PC1 station scores (Fig. 4). The modeled area, which is bounded by the locations of stations, is mostly unaltered (PC1 scores>−0.4 that are color coded as green or yellow), except for a small area (19 km^2^) near where the Mississippi River enters the Gulf of Mexico.

**Figure 5:**
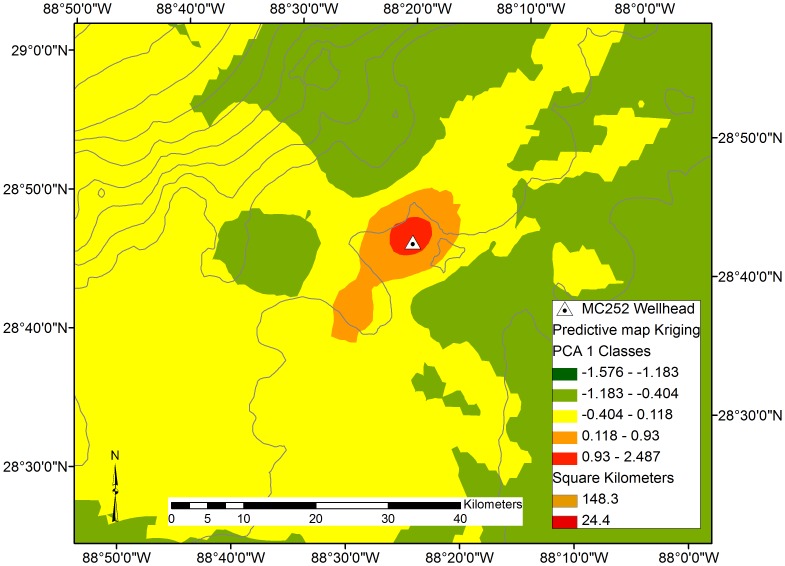
Interpolated area of deep sea impact based on PC1 station scores. The interpolated area of the zoomed in map covers 6,350 km^2^ of which 148 km^2^ (orange) are considered moderately impacted and 24 km^2^ (red) are considered severely impacted.

Zooming in, the interpolated pattern of the area of strong impacts (i.e., PC1 scores ranging from 0.931–2.487) is circular in shape and covers an area of 24.4 km^2^ (Fig. 5). The shape of the interpolated area with moderate impacts (i.e., PC1 scores ranging from 0.118–0.930) is elongated along the northeast-southwest axis and covers an area of 148 km^2^. The shape of the moderate impact area is asymmetrical, extending further to the southwest (about 17 km from the wellhead) than to the northeast (about 8.5 km from the wellhead). The 148 km^2^ area classified as moderate impacts does not include the shallowest area nearest the location where the Mississippi River enters the Gulf of Mexico (Fig. 4).

There are many natural seep features in the Gulf of Mexico (Fig. 2), and several surround the DWH wellhead site (Fig. 3). PC1 is highly correlated with distance from the wellhead, but not with distance from seeps or water depth (Table 1). PC2, is strongly correlated with water depth, implying it represents effects due to water depth. PC2 is also correlated with distance from the wellhead. PC3, which represents the relationship between sediment types and macrofauna abundance, is correlated to distance from natural seeps.

In support of the above PCA approach, a multiple linear regression (MLR) analysis also was performed to evaluate benthic impacts using abiotic variables to explain patterns in biotic variables and the significance and direction of their associations. Accordingly, the MLR model used macrofauna abundance, meiofauna abundance, macrofauna diversity, meiofauna diversity, and N:C as dependent variables and Al, Ba, PAH, TPH, and mud content as the independent variables. The MLR was run on the same transformed and standardized data as the PCA so that outliers would not distort the statistical tests. Results indicate that the driver of biotic change is PAH concentration. Moreover, the inverse relationships between PAH concentration and macrofauna diversity (coefficient estimate = −0.52) and meiofauna diversity (coefficient estimate = −0.76) are significant (p = 0.03 and p = 0.0002 respectively) and the positive relationship between PAH and N:C is significant (coefficient estimate = 0.83, p = 0.0002). None of the variance inflation factors (VIF, which is the reciprocal of tolerance) of the predictor variables were higher than 4.0, which is well below the common convention of 5 as a cutoff value. The results of the MLR are consistent with those derived from the above PCA approach and provide further confirmatory evidence of spill-related impacts to the soft-bottom deep benthos.

## Discussion

Diversity and community structure are often used as bioindicators of community integrity. Since its proposal [21], the N:C ratio has been regarded as a useful indicator of organic enrichment and pollution. While the N:C ratio may vary seasonally due to natural fluctuations in food availability [22] and sediment granulometry [23], it has worked well to classify impacts of pollution and organic enrichment in field and mesocosm studies [24], [25], [26]. More recently, and arguably more relevant to the current study, the N:C has worked well to classify impacts of drilling activities in the Gulf of Mexico [16]. While natural seasonal pulses of surfaced-derived primary production could elevate nematode dominance in deep-sea meiobenthic communities, it is unlikely that seasonality enhanced N:C in the region of the MC252 wellhead relative to more distant stations at the same depth and in the same general region of the Gulf of Mexico. Sediment granulometry is nearly constant at all stations investigated in the current study with >90% silt/clay, and therefore granulometry is not likely to have an effect on N:C here. Finally, prior surveys of the meiofauna community throughout the entire northern Gulf of Mexico deep sea revealed a Gulf-wide N:C mean of 5.7±1.8 across 5 replicate core samples taken from 51 stations ranging in depth from 200–3500 m [17], [27].

Strong positive correlations of N:C, PAH, TPH, and Ba indicate that contaminants are correlated to benthic community change in soft-bottom benthos, and this was reflected in positive scores on the PC1 axis (Fig. 1). The strong inverse correlations between measures of contaminants and diversity (Mei_N1 and Mac_N1) on PC1 provide additional evidence of such impacts. Together these results indicate that PC1 can be used as a new variable to depict the footprint of oil-spill impacts to the benthos and loss of ecological integrity. Thus PC1 defines the chemical and biological footprint of the oil spill.

The hydrocarbon flow rate from the DWH wellhead is estimated to have been approximately 10.1±2.0×10^6^ kg/day [3] and as much as 35% of released oil may have entered the observed deep-sea plume. Model simulations of hydrocarbon trajectories in the deep-sea indicate a potential for variable flow paths at different depths [28]. However, direct tracking of the plume and observed oxygen anomalies in the water column follow an overall trajectory to the southwest [29], [30] at depths of 1100–1200 m, concordant with predominant deep-water currents at that depth. The deep-sea oil plume was as much as 200 m thick and 2 km wide in some locations providing a potential mechanism for transfer of DWH hydrocarbons to deep-sea communities [29].

Several studies have reported on the observed oxygen anomaly in the deep-sea plume, and the data suggest hydrocarbon-mediated enrichment of indigenous bacteria within the water column [30], [31], [32]. Similar increases in bacterial abundance and gene expression have been observed in both deep-sea plume and coastal marsh investigations [31], [33], [34]. Bacterial blooms may have resulted in increased dissolved or particulate organic matter flux to deep-sea sediments, which could possibly enrich benthic communities. While there have been several coastal studies of benthic microbial dynamics [34], [35], we are not aware of any deep-sea sediment microbial studies published to date. In fact, it has already been pointed out that the initial round of studies of the DWH incident were lacking in deep-sea studies [4].

Increased N:C ratios at stations inside of 15 km from the wellhead indicate that meiofauna communities exhibited disproportionately high nematode abundance and dominance in comparison to more distant stations, which is consistent with an organic enrichment hypothesis. It is not likely that total organic carbon (TOC) is the enrichment driver because it does not vary much among the stations and did not explain variance when added to the PCA. The increase in nematode abundance relative to harpacticoid abundance may be the first evidence for a community-level trophic response to the possibility that the DWH spill enriched indigenous bacteria, which would then be available as food for deep-sea infauna. However, the total number of harpacticoids decreased where nematodes increased, and while we did not measure sedimentation, it is possible that some infauna was smothered or covered by spilled oil as well.

It is apparent that the Deepwater Horizon blow out and subsequent oil spill did adversely affect deep-sea soft-sediment benthos. How long will community recovery take? Little is known about deep-sea infaunal community recruitment rates or succession following a disturbance, especially one with lingering contamination of the substrate. In situ experiments indicate that deep-sea communities are slow to recolonize clean azoic sediments, taking on the order of years or longer [36]. Full recovery at impacted stations will require degradation or burial of DWH-derived contaminants in combination with naturally slow successional processes. Oil degradation in the marine environment is limited by temperature, nutrient availability (especially nitrogen and phosphorous), biodegradability of the petroleum hydrocarbons, presence of organic carbon, and the presence of microorganisms with oil degrading enzymes [37], [38]. Recovery of soft-bottom benthos after previous shallow-water oil spills has been documented to take years to decades [39], [40]. In the deep-sea, temperature is uniformly around 4°C, and TOC and nutrient concentrations are low, so it is likely that hydrocarbons in sediments will degrade more slowly than in the water column or at the surface. Also, metabolic rates of benthos in the deep-sea are very slow and turnover times are very long [41], [42]. Given deep-sea conditions, it is possible that recovery of deep-sea soft-bottom habitat and the associated communities in the vicinity of the DWH blowout will take decades or longer.

## Supporting Information

Table S1 (XLSX)

## References

[1] Griffiths SK (2012) Oil release from Macondo Well MC252 following the Deepwater Horizon Accident. Environ Sci Technol 46: 5616–5622. 22506853 10.1021/es204569t

[2] NOAA USGS(2010) BP Deepwater Horizon Oil Budget: What Happened to the Oil? www.noaanews.noaa.gov/stories2010/PDFs/OilBudget_description_%2083final.pdf.

[3] Ryerson TB, Camilli R, Kessler JD, Kujawinski EB, Reddy CM, el al (2012) Chemical data quantify Deepwater Horizon hydrocarbon flow rate and environmental distribution. Proc Natl Acad Sci U S A 109: 20246–20253. 22233807 10.1073/pnas.1110564109 PMC3528560

[4] Peterson CH, Anderson SS, Cherr GN, Ambrose RF, Anghera S, et al (2012) A tale of two spills: novel science and policy implications of an emerging new oil spill model. Bioscience 62: 461–469.

[5] UAC (Unified Area Command) (2010) Deepwater Horizon MC 252 Response Unified Area Command – Strategic Plan for Sub-Sea and Sub-Surface Oil and Dispersant Detection, Sampling, and Monitoring. November 13, 2010 Final, U.S. Coast Guard and BP Exploration and Production, Inc. New Orleans, LA. USA.

[6] Neff JM, Rabalais NN, Boesch DF (1987) Offshore oil and gas development activities potentially causing long-term environmental effects, in *Long-Term Environmental Effects of Offshore Oil and Gas Development*, Boesch DF, Rabalais NN (eds.) Elsevier Applied Science, London, 149–174.

[7] Kennicutt MCII, Boothe PN, Wade TL, Sweet ST, Rezak R, et al (1996) Geochemical patterns in sediments near offshore production platforms. Can J Fish Aquat Sci 53: 2554–2566.

[8] Danovaro R, Gambi C, Anno AD, Corinaldesi C, Fraschetti S, et al (2008) Exponential decline of deep-sea ecosystem functioning linked to benthic biodiversity loss. Current Biol 18: 1–8. 10.1016/j.cub.2007.11.056 18164201

[9] Tyler PA, Ed. (2003) Ecosystems of the Deep. Elsevier Science, Amsterdam, Netherlands.

[10] Hessler RR, Sanders HL (1967) Faunal diversity in the deep sea. Deep-Sea Res 14: 65–78.

[11] Etter RJ, Rex MA, Chase MC, Quattro JM (1999) A genetic dimension to deep-sea biodiversity. Deep-Sea Res I 46: 1095–1099.

[12] Grassle JF, Maciolek NJ (1992) Deep-sea species richness: Regional and local diversity estimates from quantitative bottom samples. Am Nat 139: 313–341.

[13] Rex MA (1981) Community structure in the deep-sea benthos. Ann Rev Ecol System 12: 331–353.

[14] Haedrich RL, Devine J, Kendal V (2008) Predictors of species richness in the deep-benthic fauna of the northern Gulf of Mexico. Deep-Sea Res II 55: 2650–2656.

[15] Wei C, Rowe GT, Hubbard GF, Scheltema AH, Wilson GDF, et al (2010) Bathymetric zonation of deep-sea macrofauna in relation to export of surface phytoplankton production. Mar Ecol Prog Ser 399: 1–14.

[16] Kennicutt MCII, Green RH, Montagna PA, Roscigno PF (1996) Gulf of Mexico Offshore Operations Experiment (GOOMEX) Phase I: Sublethal responses to contaminant exposure introduction and overview. Can J Fish Aquat Sci 53: 2540–2553.

[17] Montagna PA, Harper DE Jr (1996) Benthic infaunal long-term response to offshore production platforms in the Gulf of Mexico. Can J Fish Aquat Sci 53: 2567–2588.

[18] Hill MO (1973) Diversity and evenness: a unifying notation and its consequences. Ecology 54: 427–432.

[19] Ludwig JA, Reynolds JF (1988) Statistical Ecology. John Wiley and Sons, New York.

[20] Jenks GF (1967) The Data Model Concept in Statistical Mapping. Internat. Yearbook Cartography 7: 186–190.

[21] Raffaelli DG, Mason CF (1981) Pollution monitoring with meiofauna, using the ratio of nematodes to copepods. Mar Pollut Bull 12: 158–163.

[22] Coull BC, Hicks GRF, Wells JBJ (1981) Nematode/Copepod ratios for monitoring pollution: A rebuttal. Mar Pollut Bull 12: 278–281.

[23] Warwick RM (1981) The nematode/copepod ratio and its use in pollution ecology. Mar Pollut Bull 12: 329–333.

[24] Marcotte BM, Coull BC (1974) Pollution, diversity and meiobenthic communities in the north Adriatic (Bay of Piran; Yugoslavia). Vie Milieu 24: 281–300.

[25] Shiells GM, Anderson KJ (1985) Pollution monitoring using the nematode/copepod ratio. A practical application. Mar Pollut Bull 16: 62–68.

[26] Gee MJ, Warwick RM, Schaanning M, Berge JA, Ambrose WG (1985) Effects of organic enrichment on meiofaunal abundance and community structure in sublittoral soft sediments. J Exp Mar Biol Ecol 91: 247–262.

[27] Baguley JG, Montagna PA, Hyde LJ, Kalke RD, Rowe GT (2006) Metazoan meiofauna abundance in relation to environmental variables in the northern Gulf of Mexico deep sea. Deep Sea Res I 53: 1344–1362.

[28] Weisberg RH, Zheng L, Liu Y (2011) Tracking subsurface oil in the aftermath of the Deepwater Horizon well blowout. Geophys Monogr Ser 195: 205–215.

[29] Camilli R, Reddy CM, Yoerger DR, Van Mooy BAS, Jakuba MV, et al (2010) Tracking hydrocarbon plume transport and biodegradation at Deepwater Horizon. Science 330: 201–204. 20724584 10.1126/science.1195223

[30] Kessler JD, Valentine DL, Redmond MC, Du M, Chan EW, et al (2011) A persistent oxygen anomaly reveals the fate of spilled methane in the deep Gulf of Mexico. Science 331: 312–315. 21212320 10.1126/science.1199697

[31] Hazen TC, Dubinsky EA, DeSantin TZ, Andersen GL, Picenco YM, et al (2010) Deep-sea oil plume enriches indigenous oil-degrading bacteria. Science 330: 204–208. 20736401 10.1126/science.1195979

[32] Valentine DL, Mezić I, Maćešić S, Črnjarić-Žic N, Ivić S, et al (2012) Dynamic autoinoculation and the microbial ecology of a deep water hydrocarbon irruption. Proc Natl Acad Sci U S A 109: 20286–20291. 22233808 10.1073/pnas.1108820109 PMC3528554

[33] Lu Z, Deng Y, Van Nostrand JD, He Z, Voordeckers J, et al (2012) Microbial gene functions enriched in the Deepwater Horizon deep-sea oil plume. ISME J 6: 451–460. 21814288 10.1038/ismej.2011.91 PMC3260509

[34] Beazley MJ, Martinez RJ, Rajan S, Powell J, Piceno YM, et al (2012) Microbial Community Analysis of a Coastal Salt Marsh Affected by the Deepwater Horizon Oil Spill. PLoS ONE 7(7): e41305. 22815990 10.1371/journal.pone.0041305 PMC3399869

[35] Bik HM, Halanych KM, Sharma J, Thomas WK (2012) Dramatic Shifts in Benthic Microbial Eukaryote Communities following the Deepwater Horizon Oil Spill. PLoS ONE 7(6): e38550 doi:10.1371/journal.pone.0038550 22701662 10.1371/journal.pone.0038550 PMC3368851

[36] Grassle JF (1977) Slow recolonisation of deep-sea sediment. Nature 265: 618–619.

[37] Das N, Chandran P (2011) Microbial degradation of petroleum hydrocarbon contaminants: An overview. Biotechnol Res Internat 2011: Article ID 941810, 13 pages, doi:10.4061/2011/941810. 10.4061/2011/941810 PMC3042690 21350672

[38] Beolchini F, Rocchetti L, Regoli F, Dell’Anno A (2010) Bioremediation of marine sediments contaminated by hydrocarbons: Experimental analysis and kinetic modeling. J Hazardous Materials 182: 403–407. 10.1016/j.jhazmat.2010.06.047 20609514

[39] Boucher G (1985) Long term monitoring of meiofauna densities after the Amoco Cadiz oil spill. Mar Pollut Bull 16: 328–333.

[40] Dauvin JC (1998) The fine sand *Abra alba* community of the Bay of Morlaix twenty years after the Amoco Cadiz oil spill. Mar Pollut Bull 36: 669–676.

[41] Baguley JG, Montagna PA, Hyde LJ, Rowe GT (2008) Metazoan meiofauna biomass, grazing, and weight-dependent respiration in the Northern Gulf of Mexico deep sea. Deep-Sea Res II 55: 2607–2616.

[42] Rowe GT, Wei C, Nunnally C, Haedrich R, Montagna P, et al (2008) Comparative biomass structure and estimated carbon flow in food webs in the deep Gulf of Mexico. Deep-Sea Res II 55: 2699–2711.

